# A comparison between pylorus-preserving and distal gastrectomy in surgical safety and functional benefit with gastric cancer: a systematic review and meta-analysis

**DOI:** 10.1186/s12957-020-01910-y

**Published:** 2020-07-08

**Authors:** Xinyu Mao, Xinlei Xu, Hua Zhu, Chunpeng Ji, Xu Lu, Baolin Wang

**Affiliations:** Department of General Surgery, The Second Affiliated Hospital of Nanjing Medical University, 121 Jiangjiayuan Road, Nanjing, 210011 Jiangsu China

**Keywords:** Pylorus-preserving gastrectomy, Distal gastrectomy, Early gastric cancer, Meta-analysis

## Abstract

**Background:**

Due to better functional outcomes, pylorus-preserving gastrectomy (PPG) has been widely applied for early gastric cancer (EGC) patients as an alternative to distal gastrectomy (DG). However, controversies still persist regarding the surgical efficacy and oncological safety of PPG.

**Methods:**

Original studies comparing PPG and DG for EGC were searched in PubMed, Embase, and the Cochrane Register of Controlled Trials up to December 2019. The weight mean difference, standardized mean difference, or odds risk was used to calculate the short-term and long-term outcomes between the two groups.

**Results:**

Twenty-one comparative studies comprising 4871 patients (1955 in the PPG group and 2916 in the DG group) were enrolled in this systematic review and meta-analysis. PPG showed longer hospital day, decreased harvested lymph nodes, and more delayed gastric emptying. However, PPG had the benefits of lower incidence of anastomosis leakage, early dumping syndrome, gastritis and bile reflux, and better recovery of total protein, albumin, hemoglobin, and weight. No difference was found in operative time, blood loss, and overall complications. Moreover, the long-term survival and recurrence rate were similar in two groups.

**Conclusion:**

Owing to the non-inferiority of surgery and oncology outcomes and the superiority of function outcomes in PPG, we revealed that PPG can be clinically applicable instead of DG in EGC. However, more high-quality comparative studies and randomized clinical trials would be required for further confirmation.

## Background

The development of cancer screening programs and the popularization of endoscopic techniques have allowed the increasing proportion of early gastric cancer (EGC), particularly in Korea and Japan [1, 2]. Radical gastrectomy with D2 lymphadenectomy was recommended as the gold standard treatment for EGC, and distal gastrectomy (DG) is one such conventional surgical procedure. Due to the excellent oncological outcomes of early gastric cancer, restoration of stomach function and better postoperative quality of life were recognized as important as the radical curability of primary tumor. Pylorus-preserving gastrectomy (PPG), which is considered as a representative example of function-preserving gastrectomy, had become an alternative to distal gastrectomy for the treatment of EGC. Since the first application of PPG in 1967 [3], this approach has been introduced as a minimally invasive surgery and even extended to combine with laparoscopic technique. Moreover, the retainment of pyloric cuff and vagal nerve in PPG provided advantages such as ameliorating postoperative gastritis, bile reflux, early dumping syndromes, and improving nutritional status [4, 5]. In PPG, the infra-pyloric lymph nodes (LNs) are routinely dissected with preserving the infra-pyloric vessels, and the supra-pyloric LNs are usually omitted to preserve the right gastric artery and the hepatic branch of the vagal nerve [6, 7]. However, technical difficulty and incomplete lymph resection, which raise concerns about compromising long-term survival, contribute to the restriction on extensive application of PPG.

Although many studies comparing PPG and DG in terms of surgical and functional outcomes have been published [7–9], whether PPG is better than DG for EGC without compromising oncological safety remains debatable because of lacking long-term oncologic outcomes and high-level evidence of randomized clinical trials (RCTs). The first meta-analysis, which was published in 2014 [10], demonstrated that PPG had superior benefits in terms of lower incidence rates of early dumping syndrome, gastritis, and bile reflux as well as regaining of weight. According to the Japanese Gastric Cancer Treatment Guidelines and considerable newly published studies with relatively comprehensive data [5, 7, 11, 12], indications as well as standardized management and treatment for PPG have been well established. Therefore, we performed an updated meta-analysis to demonstrate the surgery efficacy, oncologic safety, and function recovery of PPG.

## Methods

### Literature search

A systematic literature search was carried out up to December 2019 using the following databases: PubMed, Embase, and the Cochrane Register of Controlled Trials (CENTRAL). The searches were limited to studies published in English. The search terms were as follows: “gastric cancer or stomach cancer or stomach neoplasm or gastric neoplasm” and “pylorus-preserving or pylorus preserving or function-preserving or function preserving”. Two independent reviewers (Xinyu Mao and Xinlei Xu) carefully explored related citations of the retrieved reports to prevent potential additional articles from overlooking. This meta-analysis was conducted according to the Preferred Reporting Items for Systematic Reviews and Meta-Analyses (PRISMA) statement [13].

### Inclusion and exclusion criteria

Inclusion criteria are as follows: (1) studies of patients with pathologically confirmed early gastric cancer, (2) compared pylorus-preserving gastrectomy with distal gastrectomy, (3) both open and laparoscopic procedure, (4) any kind of comparative studies, (5) revealed adequate data of the surgical or functional outcomes.

Exclusion criteria are as follows: (1) overlapped publications or duplicated data; (2) reviews, case reports, comments, and conference abstracts; (3) not addressing the comparison between pylorus-preserving gastrectomy and distal gastrectomy; (4) not relevant or available data of target endpoints.

### Data extraction and quality assessment

Two authors independently conducted the data extraction among all enrolled studies; including (1) study characteristics (authors, year, country, study design, sample size); (2) patient characteristics (age, sex, ASA, BMI, tumor size, tumor stage, tumor location, reconstruction type, proximal and distal resection margin); (3) surgical outcomes (operation time, blood loss, hospitalization day, examined lymph nodes) and postoperative complications (such as anastomosis leakage, bile reflux, gastritis, delayed gastric emptying, dumping syndrome, and gallbladder stones); (4) functional status (total protein, albumin, body weight, and hemoglobin) and long-term oncological outcomes (survival and recurrence rates). Any disagreements were resolved with discussion in conference by two independent researchers.

The Newcastle-Ottawa Quality Assessment Scale (NOS) checklist (Additional file 1), which consisted of three categories (selection, comparability, and outcome) and eight elements with a maximum score of nine, was used to evaluate the quality of enrolled observational research. Studies graded with 7 or above were considered as high-quality.

### Statistical analysis

Odds ratio (OR), weight mean difference (WMD), and standardized mean difference (SMD) presented with 95% confidence interval (CI) were used to pool analysis dichotomous and continuous variables, respectively. When the reports included in our work only report mean and range, standard deviation (SD) was estimated based on the formulas reported by Hozo et al. [14]. Overall survival (OS) was measured with the hazard ratio (HR) and 95% CI which is calculated by Engauge Digitizer Version 4.1 according to the Kaplan-Meier survival curves.

Heterogeneity was assessed by the chi-squared-based Q test and Higgins I-squared test among studies. According to the high heterogeneity with *I*^2^ > 50% or *P* < 0.1, random effects model was chosen. On the contrary, fixed effects model was preferred in terms of appreciable heterogeneity. According to the study characteristics, we performed the subgroup analyses to explore the potential cause of the heterogeneity. Funnel plots and Egger’s test were used for evaluation of publication bias. We used trim-and-fill test to estimate the influence on the results of public bias. All analyses were carried out with the Stata software (version 15; Stata Corp LLC, College Station, TX).

## Results

### Literature search

**Fig. 1:**
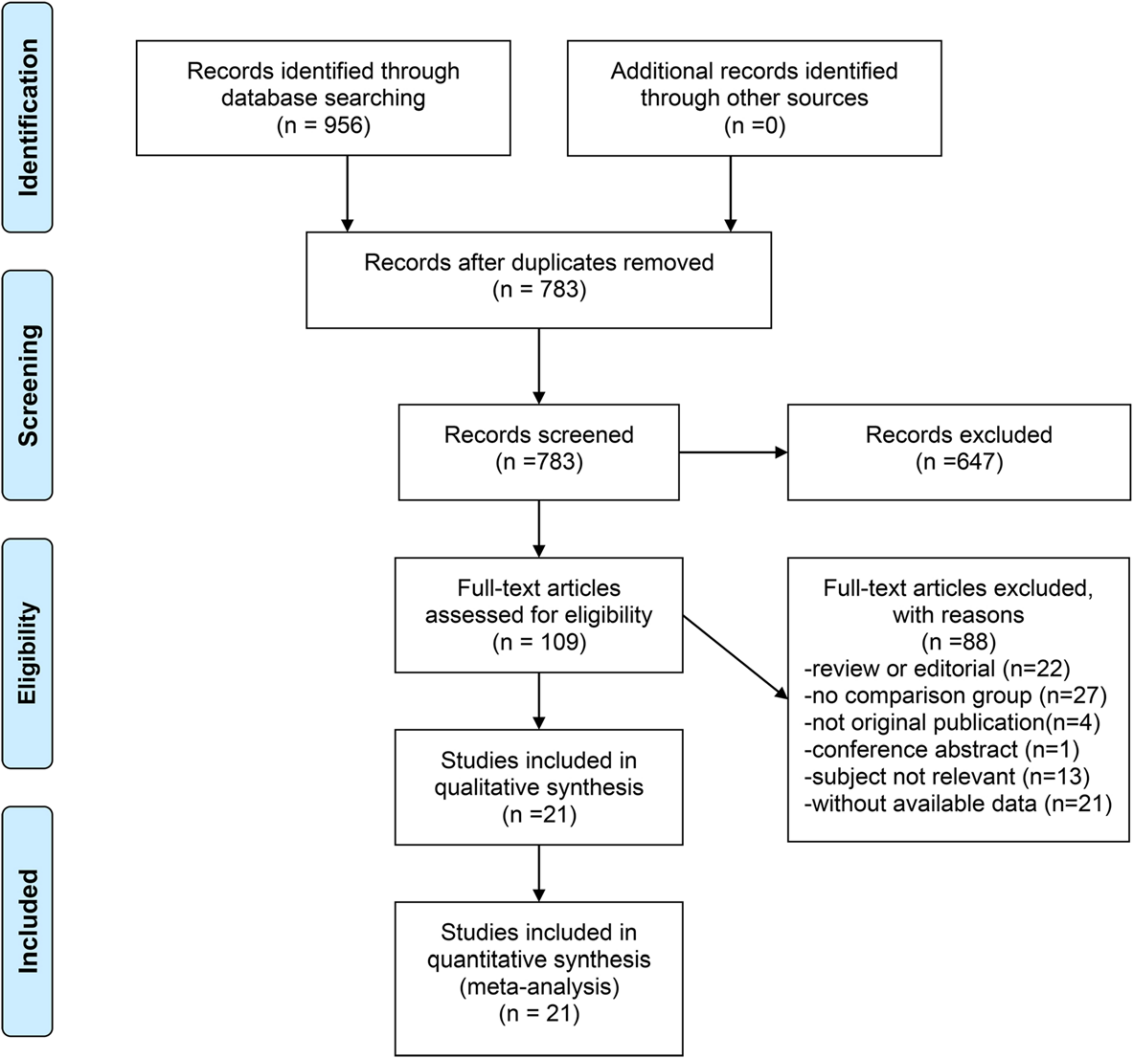
PRISMA diagram showing criteria for inclusion and exclusion

A total of 956 studies were identified in accordance with the Preferred Reporting Items for Systematic Reviews and Meta-Analyses (PRISMA) flow diagram (Fig. 1). After removing 173 duplicates, 647 non-relevant studies were initially excluded by carefully screening the title and or abstract, and subsequently 109 articles were evaluated for eligibility via cautiously reviewing full-text and statistical data. Finally, one RCT and 20 non-RCTs [4–9, 12, 15–28] with 4871 patients were included in the quantitative synthesis.

### Study characteristics

The details of these 21 comparative studies included in the meta-analysis are shown in Table 1. Among enrolled studies, 7 were performed laparoscopically, and open surgery was operated in the other fourteen studies, of which 96.9% patients were pathologically diagnosed with stage I gastric cancer. Sixteen papers were reported by Japan, and only 5 studies were published by Korea and China.

**Table 1:** Details of the articles included in the meta-analysis

References	Approach	Age	Sex (m/f)	BMI	Tumor size	Stage			Anastomosis	Nerve^a^	Antral cuff (cm)^b^	Preservation of LN station 5^c^
						I	II	III				
Park et al. [4]	CDG 17	56.2 ± 9.4	13/4	NA	NA	17	0	0	BI			
	PPG 22	57.3 ± 9.5	18/4	NA	NA	22	0	0	Gastro-gastro	Hepatic, pyloric	3	No
Zhu et al. [5]	CDG 61	NA	34/27	NA	NA	56	4	1	BII 24 + RY 37			
	PPG 145	NA	67/78	NA	NA	138	4	3	Gastro-gastro	NA	3	Yes
Imada et al. [6]	CDG 25	NA	NA	NA	NA	NA	NA	NA	NA			
	PPG 20	NA	NA	NA	NA	NA	NA	NA	NA	Hepatic, pyloric	1.5	Yes
Xia et al. [7]	LADG 97	57.5 ± 12.1	63/34	22.7 ± 4.8	1.8 ± 0.7	97	0	0	BI			
	LAPPG 70	56.8 ± 10.9	46/24	22.3 ± 2.3	1.8 ± 0.7	70	0	0	Gastro-gastro	Hepatic, pyloric	3	Yes
Shibata et al. [8]	CDG 38	60 ± 2	25/13	NA	NA	36	0	0	BI			
	PPG 36	64 ± 1	23/13	NA	NA	38	0	0	Gastro-gastro	NA	1.5	No
Suh et al. [9]	LADG 176	59.1 ± 12.0	107/69	24.0 ± 3.1	2.3 ± 1.2	166	10	0	BI 162 + BII13 + RY 1			
	LAPPG 116	54.1 ± 12.3	55/61	23.3 ± 3.0	2.6 ± 1.2	108	7	1	Gastro-gastro	NA	NA	NA
Isozaki et al. [15]	CDG 14	52.7	11/3	NA	NA	NA	NA	NA	NA			
	PPG 15	57.3	10/5	NA	NA	NA	NA	NA	Gastro-gastro	Hepatic, pyloric	1.5	Yes
Zhang et al. [16]	CDG 28	58.0 ± 17.1	21/7	NA	NA	28	0	0	BI			
	PPG 15	58.9 ± 9.4	11/4	NA	NA	15	0	0	Gastro-gastro	Hepatic, pyloric, celiac	1.5	No
Hotta et al. [17]	CDG 45	NA	NA	NA	NA	45	0	0	BI			
	PPG 19	NA	NA	NA	NA	19	0	0	Gastro-gastro	Hepatic, pyloric	1.5	Yes
Tomita et al. [18]	CDG 22	NA	NA	NA	NA	NA	NA	NA	NA			
	PPG 10	NA	NA	NA	NA	NA	NA	NA	NA	Hepatic, pyloric	1.5	No
Urushihara et al. [19]	LADG 26	68	15/11	NA	NA	26	0	0	NA			
	LAPPG 26	68	14/12	NA	NA	25	1	0	Gastro-gastro	Hepatic, pyloric	3	NA
Nunobe et al. [20]	CDG 203	NA	127/76	NA	NA	NA	NA	NA	BI			
	PPG 194	NA	121/73	NA	NA	NA	NA	NA	Gastro-gastro	Hepatic, pyloric, celiac	2.5–6.0	No
Ikeguchi et al. [21]	CDG 87	64.2	56/31	NA	3.1	79	6	2	BI			
	PPG 46	62.8	24/22	NA	2.6	46	0	0	Gastro-gastro	Hepatic, pyloric	3	Yes
Lee et al. [22]	CDG 305	NA	NA	NA	NA	NA	NA	NA	NA			
	PPG 148	NA	NA	NA	NA	NA	NA	NA	NA	NA	4	NA
Tomikawa et al. [23]	LADG 12	68.7 ± 4.8	6/6	BW	2.6	NA	NA	NA	Gastroduodenal			
	LAPPG 9	69.2 ± 6.9	6/3	NA	3.9	NA	NA	NA	Gastro-gastro	Hepatic, pyloric, celiac	3	Yes
Fujita et al. [24]	CDG 909	61.6 ± 9.1	594/311	22.7 ± 3.0	NA	NA	NA	NA	BI			
	PPG 313	61.5 ± 8.7	183/126	22.7 ± 3.0	NA	NA	NA	NA	Gastro-gastro	NA	NA	NA
Isozaki et al. [25]	CDG 21	61.0 ± 10.0	17/8	NA	3.2 ± 1.6	25	0	0	BI 21 + RY 4			
	PPG 15	61.2 ± 12.4	6/9	NA	2.5 ± 9.9	15	0	0	Gastro-gastro	Hepatic, pyloric, celiac	4	NA
Aizawa et al. [26]	CDG 502	61.7 ± 11.4	309/193	22.6 ± 3.08	27.3 ± 12.5	502	0	0	BI 334 + BII 2 + RY 166			
	PPG 502	60.7 ± 9.6	301/201	22.7 ± 3.14	27.4 ± 12.7	502	0	0	Gastro-gastro	NA	NA	NA
Hosoda et al. [27]	LADG 32	63.2 ± 8.8	13/19	22.5 ± 3.2	2.8 ± 1.7	31	0	1	NA			
	LAPPG 32	64.0 ± 9.5	13/19	22.7 ± 2.6	2.9 ± 1.5	30	2	0	NA	Hepatic, pyloric, celiac	4	Yes
Eom et al. [28]	LADG 195	56.5 ± 11.8	114/81	24.0 ± 3.1	2.8 ± 1.5	195	0	0	BII			
	LAPPG101	58.3 ± 12.0	54/47	24.1 ± 3.1	2.5 ± 1.4	101	0	0	Gastro-gastro	Hepatic, pyloric	3–5	Yes
Tsujiura et al. [12]	LADG 101	59.4 ± 11.1	72/29	27.0 ± 2.3	NA	NA	NA	NA	BI 43 + RY 58			
	LPPG 101	59.5 ± 9.3	71/30	27.1 ± 2.3	NA	NA	NA	NA	Gastro-gastro	Hepatic, pyloric	NA	Yes

*LADG* laparoscopic-assisted distal gastrectomy, *LAPPG* laparoscopic-assisted pylorus-preserving gastrectomy, *CDG* conventional distal gastrectomy, *BI* Billroth I, *BII* Billroth II, *R-Y* Roux-en-Y, *Gastro-gastro* gastrogastrostomy, *NA* not applicable

^a^Preservation of the branches of vagus nerve in pylorus-preserving gastrectomy (PPG)

^b^Length of the antral cuff in PPG

^c^Preservation of lymph nodes (LN) station 5 in PPG

Additional file 2 showed the analysis of demographic characteristics. The gender distribution in the PPG group was significantly different in both groups (OR 0.83, 95% CI 0.73 to 0.94, *I*^2^ = .00%, *P* = 0.005). No difference in articles comparing age (WMD 0.19, 95% CI − 1.71 to 2.09, *I*^2^ = 87.90%, *P* = 0.845) and BMI was demonstrated between the two groups (WMD − 0.02, 95% CI − 0.24 to 0.19, *I*^2^ = 0.00%, *P* = 0.828). What’s more, similar tumor size was observed in enrolled studies (WMD 0.02, 95% CI − 0.09 to 0.13, *I*^2^ = 31.50%, *P* = 0.767). Eighteen reports which were identified as high-quality studies graded with NOS were included in this meta-analysis and NOS scores were presented in Additional file 3.

### Intraoperative and postoperative findings

**Fig. 2:**
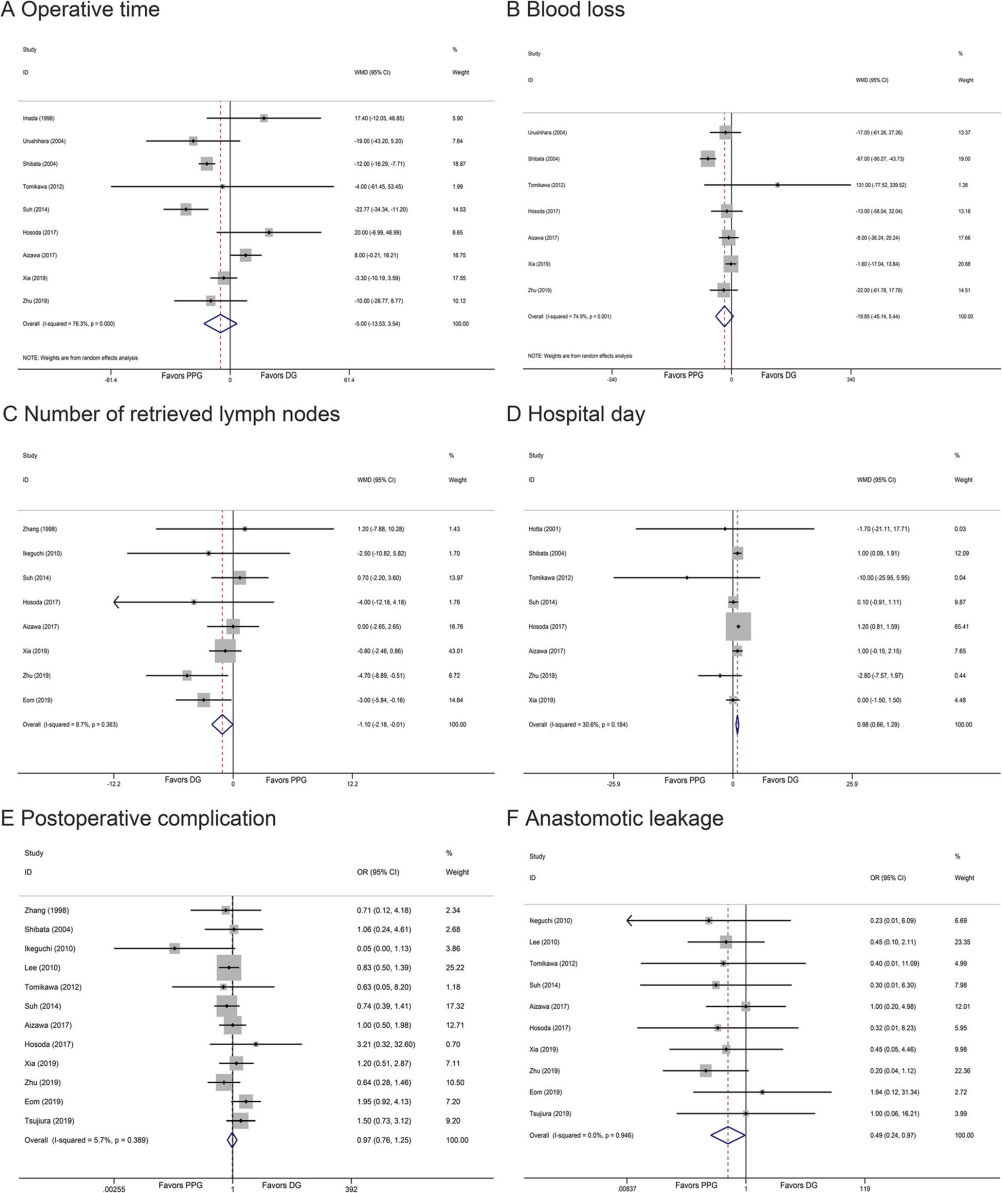
Forest plot of each outcome. **a** Operative time. **b** Blood loss. **c** Number of retrieved lymph nodes. **d** Hospital day. **e** Postoperative complication. **f** Anastomotic leakage

Table 2 showed the overall resutls between PPG and DG. Both procedures demonstrated comparative results regarding operation time (WMD − 5.00, 95% CI − 13.53 to 3.54, *I*^2^ = 76.30%, *P* = 0.251) (Fig. 2a) and blood loss (WMD − 19.85, 95% CI − 45.14 to 5.44, *I*^2^ = 74.90%, *P* = 0.124) (Fig. 2b) between the PPG and DG group. However, we observed significantly fewer retrieved lymph nodes among two groups (WMD − 1.10, 95% CI − 2.18 to − 0.01, *I*^2^ = 8.70%, *P* = 0.048) (Fig. 2c), which was also found between patients with the dissection No.5 lymph nodes compared with those with the preservation of No.5 lymph nodes. We also found longer hospital duration in PPG group (WMD 0.98, 95% CI 0.66 to 1.29, *I*^2^ = 30.60%, *P* = 0.000) (Fig. 2d). However, the PPG group had a significantly shorter proximal resection margin and distal resection margin when compared to DG group (WMD − 0.47, 95% CI − 0.91 to − 0.03, *I*^2^ = 74.30%, *P* = 0.038; WMD − 2.76, 95% CI − 4.96 to − 0.57, *I*^*2*^ = 98.20%, *P* = 0.013).

**Table 2:** Overall results comparing PPG with DG

	No. of studies	OR/WMD (95%CI)	*P*	Heterogeneity		Effect model
				*I*^2^	*P*	
Age	12	0.19 (− 1.71, 2.09)	0.845	87.90	**0.000**	Random
Gender	17	0.83 (0.73, 0.94)	**0.005**	0.00	0.876	Fixed
BMI (kg/m^2^)	7	− 0.02 (− 0.24, 0.19)	0.828	0.00	0.587	Fixed
Tumor size (cm)	6	0.02 (− 0.09, 0.13)	0.767	31.50	0.200	Fixed
Operation time (min)	9	− 5.00 (− 13.53, 3.54)	0.251	76.30	**0.000**	Random
Blood loss (ml)	7	− 19.85 (− 45.14, 5.44)	0.124	74.90	**0.001**	Random
Proximal resection margin (cm)	4	− 0.47 (− 0.91, − 0.03)	**0.038**	74.30	**0.009**	Random
Distal resection margin (cm)	4	− 2.76 (− 4.96, − 0.57)	**0.013**	98.20	**0.000**	Random
Retrieved lymph nodes	8	− 1.10 (− 2.18, 0.01)	**0.048**	8.70	0.363	Fixed
Postoperative hospital stay (day)	8	0.98 (0.66, 1.29)	**0.000**	30.60	0.184	Fixed
Complications	12	0.97 (0.76, 1.25)	0.835	5.70	0.389	Fixed
Anastomotic leakage	10	0.49 (0.24, 0.97)	**0.041**	0.00	0.946	Fixed
Gastritis	8	0.22 (0.07, 0.74)	**0.014**	71.30	**0.001**	Random
Esophagitis	4	1.21 (0.56, 2.61)	0.621	37.90	0.185	Fixed
Bile reflux	6	0.30 (0.10, 0.89)	**0.031**	57.90	**0.037**	Random
Early dumping syndrome	4	0.18 (0.07, 0.44)	**0.000**	0.00	0.982	Fixed
Gallbladder stones	6	0.63 (0.38, 1.03)	0.063	14.60	0.320	Fixed
Gastric stasis	11	1.88 (1.23, 2.87)	**0.003**	38.40	0.093	Fixed
Gastric emptying times (50%, min)	3	8.86 (1.71, 16.00)	**0.015**	0.00	0.433	Fixed
Total protein*	7	0.39 (0.25, 0.53)	**0.000**	34.60	0.164	Fixed
Albumin*	7	0.31 (0.17, 0.44)	**0.000**	22.00	0.261	Fixed
Hemoglobin*	5	0.55 (0.39, 0.71)	**0.000**	0.00	0.576	Fixed
Body weight loss (Kg)	8	3.24 (1.79, 4.69)	**0.000**	65.30	**0.005**	Random
Recurrence	6	1.41 (0.68, 2.89)	0.355	0.00	0.932	Fixed
Overall survival rate	3	0.63 (− 0.06, 1.32)	0.074	0.00	0.951	Fixed

*P* < 0.05 are indicated in bold

*OR* odds ratio, *WMD* weight mean difference, *CI* confidence interval

*Effect size is presented by standardized mean difference (SMD)

### Morbidity and mortality

**Fig. 3:**
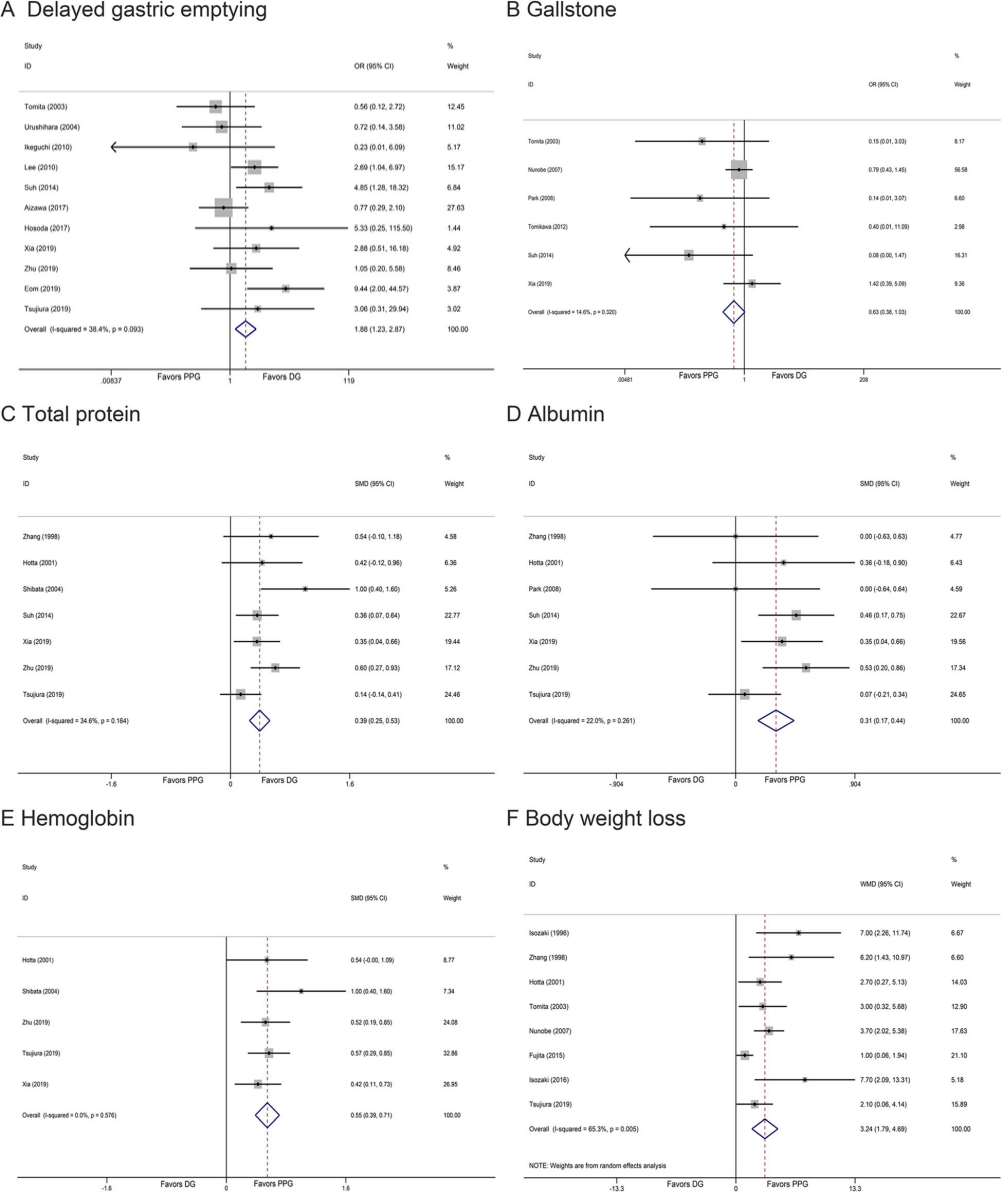
Forest plot of each outcome. **a** Delayed gastric emptying. **b** Gallstone. **c** Total protein. **d** Albumin. **e** Hemoglobin. **f** Body weight loss

This meta-analysis demonstrated that there was no significant difference in the incidence of postoperative complications (OR 0.97, 95% CI 0.76 to 1.25, *I*^2^ = 5.70%, *P* = 0.835) (Fig. 2e). Incidence rate of anastomotic leakage was significant lower in PPG (OR 0.49, 95% CI 0.24 to 0.97, *I*^2^ = 0.00%, *P* = 0.041) (Fig. 2f). In addition, there was significant difference in delayed gastric emptying (OR 1.88, 95% CI 1.23 to 2.87, *I*^2^ = 38.40%, *P* = 0.003) (Fig. 3a) between two groups, which was also confirmed by assessing gastric emptying times (WMD 8.86, 95% CI 1.71 to 16.00, *I*^2^ = 0.00%, *P* = 0.015).What’s more, PPG group revealed similar gallbladder stone rate (OR 0.63, 95% CI 0.38 to 1.03, *I*^2^ = 14.60%, *P* = 0.063) (Fig. 3b), but lower incidence of early dumping syndrome (OR 0.18, 95% CI 0.07 to 0.44, *I*^2^ = 0.00%, *P* = 0.000). In terms of postoperative endoscopic findings and symptoms (Additional file 4), patients performed with PPG procedure suffered fewer gastritis and bile regurgitation (OR 0.22, 95% CI 0.07 to 0.74, *I*^2^ = 71.30%, *P* = 0.014; OR 0.30, 95% CI 0.10 to 0.89, *I*^2^ = 57.90%, *P* = 0.031), which was not found in esophagitis (OR 1.21, 95% CI 0.56 to 2.61, *I*^2^ = 37.90%, *P* = 0.621).

### Long-term oncological and nutritional outcomes

Discrepancy was not found on the overall survival rate described by three included studies (WMD 0.63, 95% CI − 0.06 to 1.32, *I*^2^ = 0.00%, *P* = 0.074), and patients receiving PPG did not have a higher incidence of recurrence (OR 1.41, 95% CI 0.68 to 2.89, *I*^2^ = 0.00%, *P* = 0.355) (Additional file 5). As shown in Fig. 3, the serum total protein and albumin level in patients with PPG were higher compared with those with DG (SMD 0.39, 95% CI 0.25 to 0.53, *I*^2^ = 34.60%, *P* = 0.000; SMD 0.31, 95% CI 0.17 to 0.44, *I*^2^ = 22.00%, *P* = 0.000). Hemoglobin investigated in five articles showed better recovery (SMD 0.55, 95% CI 0.39 to 0.71, *I*^2^ = 0.00%, *P* = 0.000). Moreover, PPG was found a better selection with fewer decreased body weight (WMD 3.24, 95% CI 1.79 to 4.69, *I*^2^ = 65.30%, *P* = 0.000).

### Subgroup analysis

According to year of publication, study type, and operation procedure, subgroup analysis was performed to explain heterogeneity and evaluate the possible effect of these parameters (Table 3). Shorter operative time and less blood loss of PPG was only detected in the non-retrospective studies. Significant lower incident rate of gastritis in PPG group was observed in the retrospective studies, the studies with laparoscopic procedure and the studies before 2010. Higher incidence of delayed gastric emptying was found in the PPG group of retrospective and non-retrospective studies, the studies with open surgery and the studies after 2010. In terms of postoperative body weight change, we observe significant differences in all subgroup analysis.

**Table 3:** Subgroup-analysis by publication year, study design, and type of the procedure

Items	*n*		Test for overall effect		Test for heterogeneity	
		OR/WMD (95%CI)	*Z*	*P*	*I*^2^	*P*
Operative time						
Before 2010	3	− 8.22 (− 23.46, 7.01)	1.06	0.290	51.4	0.128
After 2010	6	− 3.28 (− 14.91, 8.35)	0.55	0.580	76.6	**0.001**
Retrospective cohort study	8	− 3.22 (− 13.68, 7.24)	0.60	0.547	71.9	**0.001**
Non-retrospective cohort study	1	− 12.00 (− 16.29, − 7.71)	5.48	**0.000**	NA	NA
Laparoscopic	5	− 8.08 (− 22.21, 6.05)	1.12	0.262	69.2	**0.011**
Open	4	− 1.25 (− 15.59, 13.09)	0.17	0.864	85.5	**0.000**
Blood loss						
Before 2010	2	− 45.69 (− 94.15, 2.77)	1.85	0.065	74.0	**0.050**
After 2010	5	− 5.16 (− 17.47, 7.15)	0.82	0.411	0.0	0.612
Retrospective cohort study	6	− 6.01 (− 17.87, 5.85)	0.99	0.321	0.0	0.709
Non-retrospective cohort study	1	− 67 (− 90.2, − 43.73)	5.64	**0.000**	NA	NA
Laparoscopic	4	− 3.6 (− 17.44, 10.24)	0.51	0.610	0.0	0.535
Open	3	− 33.53 (− 73.60, 6.53)	1.64	0.101	81.6	**0.004**
Gastritis						
Before 2010	7	0.22 (0.06, 0.79)	2.31	**0.021**	75.0	0.001
After 2010	1	0.23 (0.01, 6.09)	0.89	0.376	NA	NA
Retrospective cohort study	6	0.16 (0.07, 0.40)	3.98	**0.000**	13.9	0.325
Non-retrospective cohort study	2	0.88 (0.13, 5.88)	0.13	0.898	45.9	0.174
Laparoscopic	1	0.23 (0.01, 6.09)	0.89	0.376	NA	NA
Open	7	0.22 (0.06, 0.79)	2.31	**0.021**	75.0	0.001
Delayed gastric emptying						
Before2010	2	0.64 (0.21, 1.96)	0.79	0.430	0.0	0.832
After2010	9	2.26 (1.43, 3.59)	3.46	**0.001**	34.6	0.141
Retrospective cohort study	10	1.74 (1.09, 2.77)	2.31	**0.021**	41.6	0.080
Non-retrospective cohort study	1	2.69 (1.04, 6.97)	2.04	**0.041**	NA	NA
Laparoscopic	7	3.03 (1.60, 5.73)	3.41	**0.001**	26.3	0.228
Open	4	1.23 (0.69, 2.20)	0.69	0.492	32.0	0.220
Length< 3 cm	1	0.56 (0.12, 2.72)	0.72	0.474	NA	NA
Length> 3 cm	7	2.34 (1.33, 4.13)	2.94	**0.003**	28.6	0.210
Length NA	3	1.70 (0.84, 2.84)	1.48	0.138	60.3	0.080
Body weight change						
Before 2010	5	3.69 (2.53, 4.84)	6.26	**0.000**	0.0	0.429
After 2010	3	2.22 (0.02, 4.43)	1.97	**0.048**	66.5	**0.050**
Retrospective cohort study	7	3.18 (1.54, 4.83)	3.79	**0.000**	63.2	**0.012**
Non-retrospective cohort study	1	3.70 (2.02, 5.38)	4.31	**0.000**	NA	NA
Laparoscopic	1	2.10 (0.06, 4.14)	2.02	**0.043**	NA	NA
Open	7	3.58 (1.84, 5.33)	4.03	**0.000**	70.3	**0.003**

*P* < 0.05 are indicated in bold

*OR* odds ratio, *WMD* weight mean difference, *CI* confidence interval, *NA* not applicable

.

### Sensitivity and publication bias

**Fig. 4:**
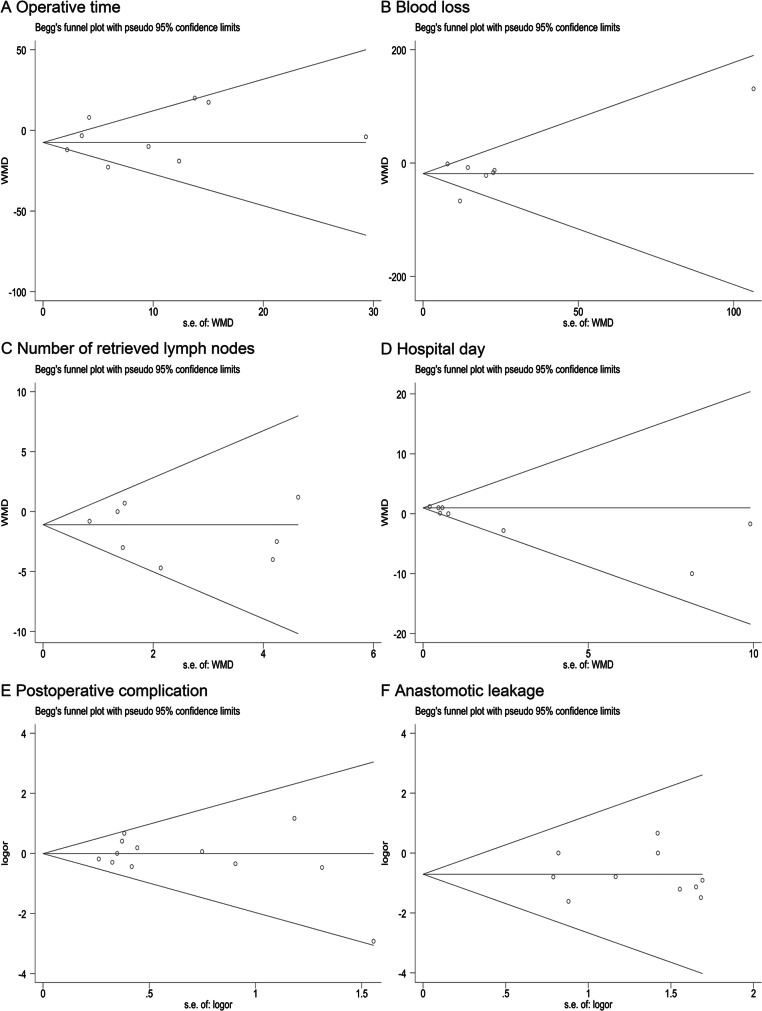
Funnel plots comparing **a** operative time, **b** blood loss, **c** number of retrieved lymph nodes, **d** hospital day, **e** postoperative complication, **f** anastomotic leakage

**Fig. 5:**
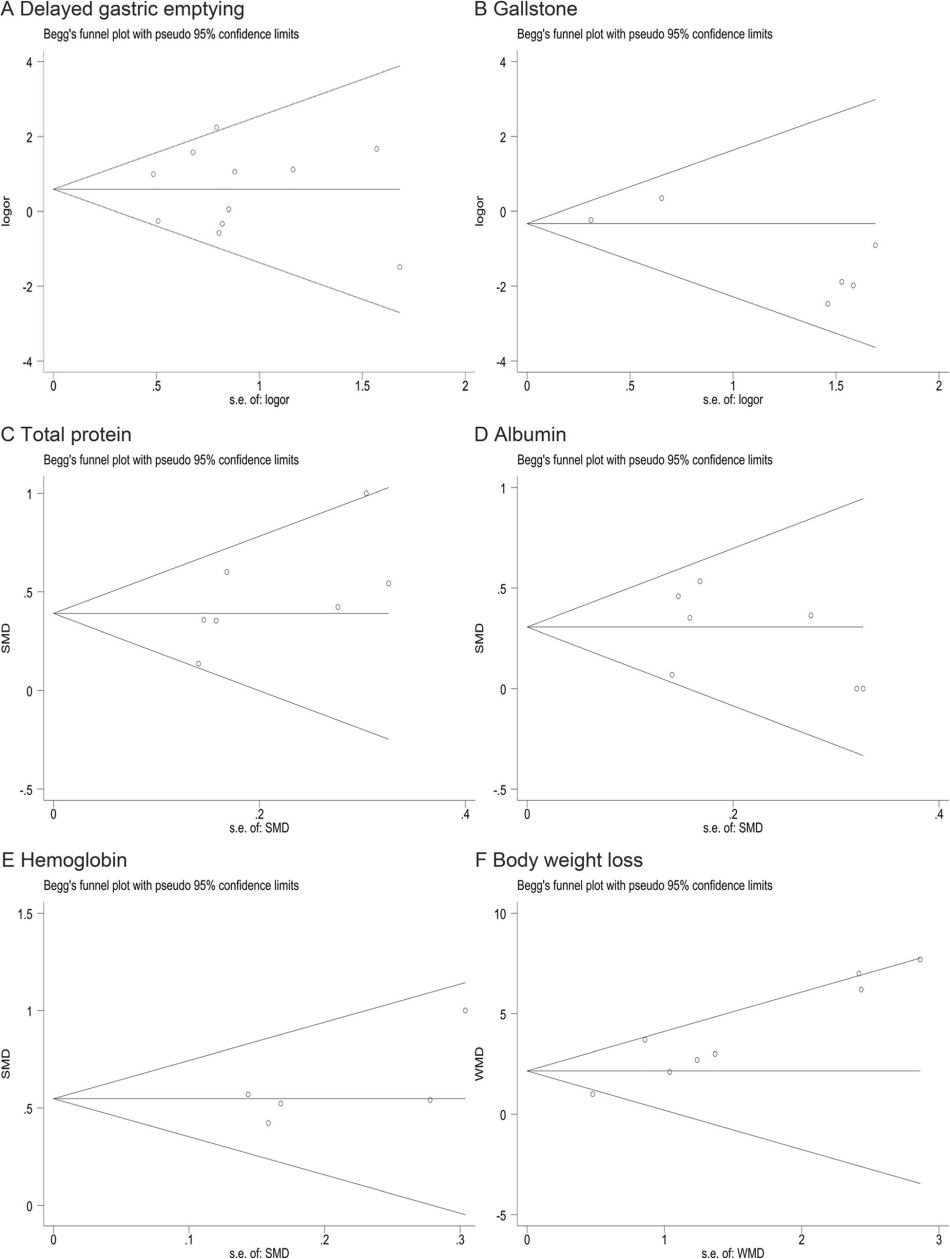
Funnel plots comparing **a** delayed gastric emptying, **b** gallstone, **c** total protein, **d** albumin, **e** hemoglobin, **f** body weight loss

Possibility of publication bias was detected with constructing funnel plots and Egger’s linear regression test. We did not find significant publication bias except for gastritis and body weight change (Fig. 4, Fig. 5 and Additional file 6). However, trim-and-fill test indicated the stability of these results. Furthermore, Galbraith plot was used to assess every individual study which account for the heterogeneity, and similar results were observed after exclusion of these researches in the supplemental information (Additional file 7, 8, 9).

## Discussion

Since the first report on the PPG for the treatment of gastric ulcer was published in 1967 [3], the indication of this procedure has been broadened to early gastric cancer [29, 30]. Moreover, this procedure was often performed with laparoscopic approach as less-invasive surgery. In addition, PPG is recommended for cT1N0M0 gastric cancer located in the middle-third of the stomach according to the Japanese Gastric Cancer Treatment Guidelines [11]. Although numerous published studies confirmed the non-inferiority of surgical outcomes and better function status of PPG, controversies still exist because of lacking high-quality RCTs. The ongoing KLASS-04 (NCT No.02595086) comparing LAPPG and LADG for EGC is expected to provide conclusive evidence. Therefore, this meta-analysis aimed to assess the surgery efficacy, oncologic safety,and function recovery of PPG.

The first meta-analysis published in 2014 included 16 studies with 1774 patients and demonstrated that PPG provided the benefits of preventing early dumping syndrome, bile reflux, and gastritis [10]. Given more additional articles published to compare PPG with DG for early gastric cancer, we accordingly performed an updated meta-analysis to corroborate surgical safety, oncological efficacy, and better function status for PPG. Moreover, depending on the published date, study design, and surgical procedure, we grouped enrolled studies to compare PPG and DG more precisely. Additionally, we matched factors such as the length of antral cuff and the vagus nerve preserved that might influence postoperative outcomes like gastritis and delayed gastric emptying (DGE). Furthermore, long-term oncological adequacy published with three studies followed for more than 3 years and function outcomes (total protein, albumin, hemoglobin, and body weight loss) were particularly evaluated on the comparison of PPG versus DG. To the best of knowledge, this is the biggest sample size meta-analysis and systematic review on PPG and DG including long-term oncological and functional outcomes up to date.

This meta-analysis presented similar operation duration and blood loss between PPG and DG. Interestingly, shorter operative time and lesser blood loss were demonstrated in PPG group in one RCT [8]. This phenomenon may be explained by the relatively few prospective articles and small sample size. However, shorter operating time and lesser blood loss may be noted with the accumulation of PPG experience, but further big-sample researches or RCTs are needed. Additionally, postoperative hospitalization is a very important indicator of recovery and hospital expenses. We found that PPG had the disadvantage of longer hospital day, which was also demonstrated in the subcategory analysis except for the prospective cohort studies. The same reason as decreased operation time and blood loss may account for exception of the prospective studies. This revealed that PPG might delay earlier recovery in both laparoscopic and open operation.

Owing to the skepticism regarding the incomplete lymphadenectomy, the oncological safety remains as the main issue, and the number of retrieved lymph nodes is related to the long-term survival. However, the harvested lymph nodes in PPG was significantly less than that in conventional DG, which can be attributed to incomplete dissection of supra-pyloric (5) and infra-pyloric (6) lymph nodes in PPG. The outcomes of the studies after 2010 and the prospective cohort studies were in favor of DG. The disparity might be explained by the difference in preserving No.5 lymph nodes. The preserving of No.5 lymph nodes was usually performed in the studies after 2010 but omitted in the studies before 2010. Furthermore, there was significant difference of dissected lymph nodes in studies with the preservation of No.5 lymph nodes. Lack of No.5 lymph node station is considered to preserve the right gastric artery and pyloric branch of vagal nerve, and preserving the infra-pyloric artery may lead to incomplete No.6 LN dissection. Given the relatively easy technique of dissecting infra-pyloric lymph nodes with infra-pyloric artery preservation, all studies enrolled in this meta-analysis from 2017 to 2019 radically resected LN station 6 but omitted LN station 5, which bring about incomplete D1 lymphadenectomy and concerns over oncological safety. Despite the significantly lower number of retrieved LNs in PPG, long-term survival rates were comparable for both groups in the meta-analysis. One possible explanation was the low metastasis rate of supra-pyloric lymph nodes in EGC. As described in a Korean report, the incidence of lymph node metastasis at the LN station 5 was 0.45% (1/220) [31]. A study of 219 cases revealed only 0.46% metastasis rate of supra-pyloric stations for gastric cancer invading mucosal or submucosal [32]. Oh et al. reported that the metastasis rate to supra-pyloric nodes was 4.2% (52/1245) [33].

According to the current version of the Japanese Gastric Cancer Treatment Guidelines [11], patients with cT1N0M0 gastric cancer located in the middle one-third of the stomach and at least 4.0 cm away from the pylorus can be candidates for PPG. This corroborates that PPG has a similar oncologic safety for intramucosal or submucosal carcinoma without on evidence of metastasis. However, Kong et al. [32] suggested that T1a and T2 cancers of the ≥ 6 DRM group showed no metastasis to LNS 5 and supported pylorus-preserving gastrectomy as a safe treatment for T2 cases with preoperatively diagnosed as T1. A retrospective study reported on better prognoses of T2 gastric cancers that were diagnosed preoperatively as T1 than the other T2 cancers [34]. Therefore, PPG may be a safe procedure for T2 cancers with no evidence of lymph metastasis. Nevertheless, further validations are needed to expand the indication for PPG in T2 cancers.

Due to preservation of the infra-pyloric vessels and hepatic branch of the vagus nerve, PPG has the advantage of better pyloric function and quality of life. Although similar postoperative overall complications were observed in both procedures, fewer anastomotic leakages were found in the PPG groups despite no significant difference was observed in the subgroup analysis. The decreased anastomotic fistula may be associated with better blood supply and function recovery. As described in previous reports [35], several risk factors such as advanced age, anemia, and malnourishment may contribute to anastomotic leakage. In our experience, reducing the anastomosis tension and ensuring the blood supply extremity have a beneficial effect on the healing of anastomosis, no matter to the patients' physical condition. For patients with PPG, higher hemoglobin level might be partly responsible for this result, as well as retaining the tissue around anastomosis and leaving the right gastric artery and the infra-pyloric artery. Thereby, PPG has a similar or even better short-term technical safety compared with DG. This should be corroborated by the final outcomes of large-scale RCTs.

Moreover, fewer patients in PPG group suffered from postoperative early dumping syndromes. The mechanisms of early dumping syndromes have yet to been confirmed. The resection of pyloric and/or vagus nerve might be associated with this phenomenon [36], and this is why there is a clear distinction between PPG patients and DG patients. However, few studies after 2010 have tried to report this complication after PPG, and a standard definition and diagnostic criteria of early dumping syndromes have not been identified. In addition, compromising hepatic and pyloric branches of the vagus nerve can increase the incidence of gallstones. In PPG, the physiological reconstruction without vagotomy maintains the contraction of Oddi sphincter and secretion of cholecystokinin [37, 38], whereas the risk of gallstones formation was decreased in the PPG group but not significantly in comparison with DG.

What’s more, the most frequent postoperative dysfunction in PPG was generally thought as DGE, and similar phenomenon was observed in our analysis. More DGE was found in the PPG group in the studies after 2010, the retrospective studies, and studies performed with laparoscopic PPG. This result may be caused by lesser extent of gastric resection, and subsequently the remaining pylorus and remnant stomach can result in postprandial fullness and dyspepsia. The influence of the antral cuff on DGE was investigated in our meta-analysis, in which studies with an antral cuff length of > 3 cm showed a significantly higher incidence of DGE, but one study with the antral cuff maintained < 3 cm did not reach a level of significance. Owing to just one article with < 3 cm antral cuff was published, the results may be influenced by the relatively small sample size. Additionally, Morita et al. retrospectively analyzed 408 patients with an antral cuff greater than 3 cm and less than 3 cm [39]. The difference among postoperative symptoms, including DGE, was not statistically significant. Moreover, comparing with patients with an antral cuff of 1.5 cm, better postoperative outcomes were found in those who had a 2.5 cm antral cuff [40]. In addition, the incidence of gastric stasis was lower with preservation of infra-pyloric blood supply in conventional pylorus-preserving gastrectomy (cPPG), which ranged from 5.7 to 8.0% reported previously [7, 9, 41, 42]. Kiyokawa et al. showed that delayed gastric emptying was found in 8.5% patients from the cPPG group but no patient in group with the preservation of infra-pyloric vein [42]. Moreover, intraoperative manual dilatation of pylorus also significantly reduced the DGE rate. The DGE rate in the cPPG group (8.6%) was significantly higher to that with manual dilatation (1.1%) among a total of 232 patients [43]. Therefore, the manual pyloric dilatation as well as preservation of infra-pyloric vessels might be an important step to minimize the most disturbing complications.

Postoperative endoscopic finding in this meta-analysis showed a favorable trend toward PPG. In the present study, the incidence of gastritis and bile regurgitation was significantly lower in PPG group, but significant difference in esophagitis was not found. For all enrolled studies, endoscopy was performed at least 6 months after the operation to evaluate esophagitis, gastritis, or bile reflux. In one retrospective study enrolled in this study with a follow-up of more than 5 years, the frequency of gastritis was significantly lower in the PPG group (10.0%) than in the DG group (63.6%), but the esophagitis frequency suggested no significant difference [18]. Park et al. demonstrated that gastritis and bile regurgitation postoperatively were only found in the DG group [4]. We think the most important factor was the reduction of the range of gastrectomy and retainment of pylorus function. The delayed gastric emptying resulting in food residue would be relevant to the development of inflammation in the remnant gastric [44]. Moreover, the chronic injury mediated by reflux of bile or gastric contents showed significant correlation with the increased incidence of Barrett’s esophagus, which is a known precursor of most esophageal adenocarcinomas [45]. Genco et al. recently reported 17.2% patients diagnosed with Barrett’s esophagus after bariatric surgery, while Barrett’s esophagus developed in 73.6% patients with reflux symptoms [46]. In the paper by Braghetto et al., Barrett’s esophagus was diagnosed between 5 and 6 years after surgery [47]. In terms of reducing the incidence of Barrett’s esophagus and the progression to esophageal adenocarcinomas, PPG has a significant advantage over DG, but a long-lasting screening and surveillance program is still necessary for early detection.

It was generally expected that PPG would improve nutrition index and decrease weight loss. For PPG cases, the recovery of total protein, albumin, and hemoglobin was better than DG cases. More extent of gastrectomy considerably reduced absorption and reservoir function of the stomach such as the secretion of gastric acid, and resection of the vagus nerve also influences the peristalsis of the stomach and duodenum. It is important that postoperative gastritis, bile regurgitation, and dumping syndrome may be associated with decreased oral intake and weight loss [17]. In the subgroup analysis, PPG leads to better function outcomes except the studies before 2010 had a similar albumin level compared with DG. For nutritional assessment, body weight loss was also a useful indictor, and the lower decreased rate of body weight was seen in our studies and subgroup analysis.

This meta-analysis had several limitations. First, given only one randomized controlled published to compare PPG and DG, most of enrolled studies were retrospective studies, and the inherent selection bias reduced the level of evidence. Second, all the participants in this meta-analysis were enrolled in the East Asia, therefore the results should be carefully generalizable to Western countries. Third, publication bias with Egger’s test was found in gastritis and body weight loss while not performed on parameters with insufficient studies. Despite performing considerable stratification analyses, we could not fully eliminate the statistical heterogeneity. Fourth, total protein, albumin, and hemoglobin were analyzed by SMD because of different assessment methods. Thus, large-scale RCTs compared with PPG and DG are needed to further corroborate these conclusions.

In conclusion, PPG is a feasible and safe option for early gastric cancer due to similar clinical effects as compared with DG. PPG has the benefits of decreasing risk of anastomotic leakage, early dumping syndrome, gastritis and bile reflux. It can prevent the deficiency of total protein, albumin and hemoglobin, and the loss of weight. However, the longer hospital day, decreased lymph node retrieval and more DGE may be the disadvantages of PPG. Moreover, the operation time, the blood loss, and the long-term survival rate showed no difference when compared to DG. These observations need to be confirmed by well-designed multicenter RCTs.

## Supplementary information

**Additional file 1.** Newcastle–Ottawa quality assessment scale.

**Additional file 2.** Forest plot of each outcome. (a) Age; (b) Sex; (c) BMI; (d) Tumor size; (e) PRM; (f) DRM; PRM, proximal resection margin; DRM, distal resection margin.

**Additional file 3.** Assessment of quality of studies.

**Additional file 4.** Forest plot of each outcome. (a) Gastritis; (b) Esophagitis; (c) Bile reflux; (d) Early dumping syndrome; (e) Time to half gastric emptying.

**Additional file 5.** Forest plot of each outcome. (a) Survival rate; (b) Recurrence.

**Additional file 6.** Funnel plots comparing (a) Gastritis, (b) Esophagitis, (c) Bile reflux, (d) Early dumping.

**Additional file 7.** Galbraith plots comparing (a) Operative time, (b) Blood loss, (c) Number of retrieved lymph nodes, (d) Hospital day, (e) Postoperative complication, (f) Anastomotic leakage.

**Additional file 8.** Galbraith plots comparing (a) Delayed gastric emptying, (b) Gallstone, (c) Total protein, (d) Albumin, (e) Hemoglobin, (f) Body weight loss.

**Additional file 9.** Galbraith plots comparing (a) Gastritis, (b) Esophagitis, (c) Bile reflux, (d) Early dumping syndrome.

**Additional file 10.** PRISIMA checklist.

## Data Availability

The datasets supporting the conclusions of this article are included within the article and its additional files.

## Abbreviations

PPG: Pylorus-preserving gastrectomy
DG: Distal gastrectomy
EGC: Early gastric cancer
LNs: lymph nodes
RCT: Randomized clinical trial
OR: Odds ratio
WMD: Weight mean difference
SMD: Standardized mean difference
CI: Confidence interval
SD: Standard deviation
HR: Hazard ratio
OS: Overall survival
DGE: Delayed gastric emptying
cPPG: Conventional pylorus-preserving gastrectomy
