# Supplementary material for: A comparison between pylorus-preserving and distal gastrectomy in surgical safety and functional benefit with gastric cancer: a systematic review and meta-analysis
Source: World J Surg Oncol. 2020 Jul 8;18:160. doi: 10.1186/s12957-020-01910-y (PMC7346397; doi:10.1186/s12957-020-01910-y)
Supplement: Supplementary file 1 — Additional file 1. Newcastle–Ottawa quality assessment scale. [file 12957_2020_1910_MOESM1_ESM.docx]

| Selection   1. Representativeness of the exposed cohort 2. Truly representative of the average “GC patients” in the community (1 star) 3. Somewhat representative of the average ‘‘GC patient’’ in the community (1 star) 4. Selected group of users eg nurses, volunteers 5. No description of the derivation of the cohort 6. Selection of the non-exposed cohort |
| --- |
| 1. Drawn from the same community as the exposed cohort (1 star) 2. Drawn from a different source 3. No description of the derivation of the non-exposed cohort 4. Ascertainment of exposure   (a) Secure record (e.g., surgical records) (1 star)  (b) Structured interview (1 star)  (c) Written self-report  (d) No description  (4) Demonstration that outcome of interest was not present at start of study  (a) Yes (1 star)  (b) No  Comparability   1. Comparability of cohorts on the basis of the design or analysis 2. Study controls for ‘‘age, sex, BMI or BW’’ (1 star)   (b) Study controls for any additional factor (1 star) (tumor location, tumor size, stage etc.)  Outcome   1. Assessment of outcome   (a) Independent blind assessment (1 star)  (b) Record linkage (1 star)  (c) Self-report  (d) No description |
| 1. Was follow-up long enough for outcomes to occur? |
| (a) Yes (‘‘2 years’’) (1 star)  (b) No |
| (3) Adequacy of follow-up of cohorts |
| (a) Complete follow-up—all subjects accounted for (1 star)  (b) Subjects lost to follow-up unlikely to introduce bias—small number lost ‘‘5 %’’ or description provided of those lost (1 star)  (c) Follow-up rate ‘‘[95 %’’ and no description of those lost  (d) No statement |

Table S1 Newcastle–Ottawa quality assessment scale
