# Supplementary figures and images for: A comparison between pylorus-preserving and distal gastrectomy in surgical safety and functional benefit with gastric cancer: a systematic review and meta-analysis
Source: World J Surg Oncol. 2020 Jul 8;18:160. doi: 10.1186/s12957-020-01910-y (PMC7346397; doi:10.1186/s12957-020-01910-y)

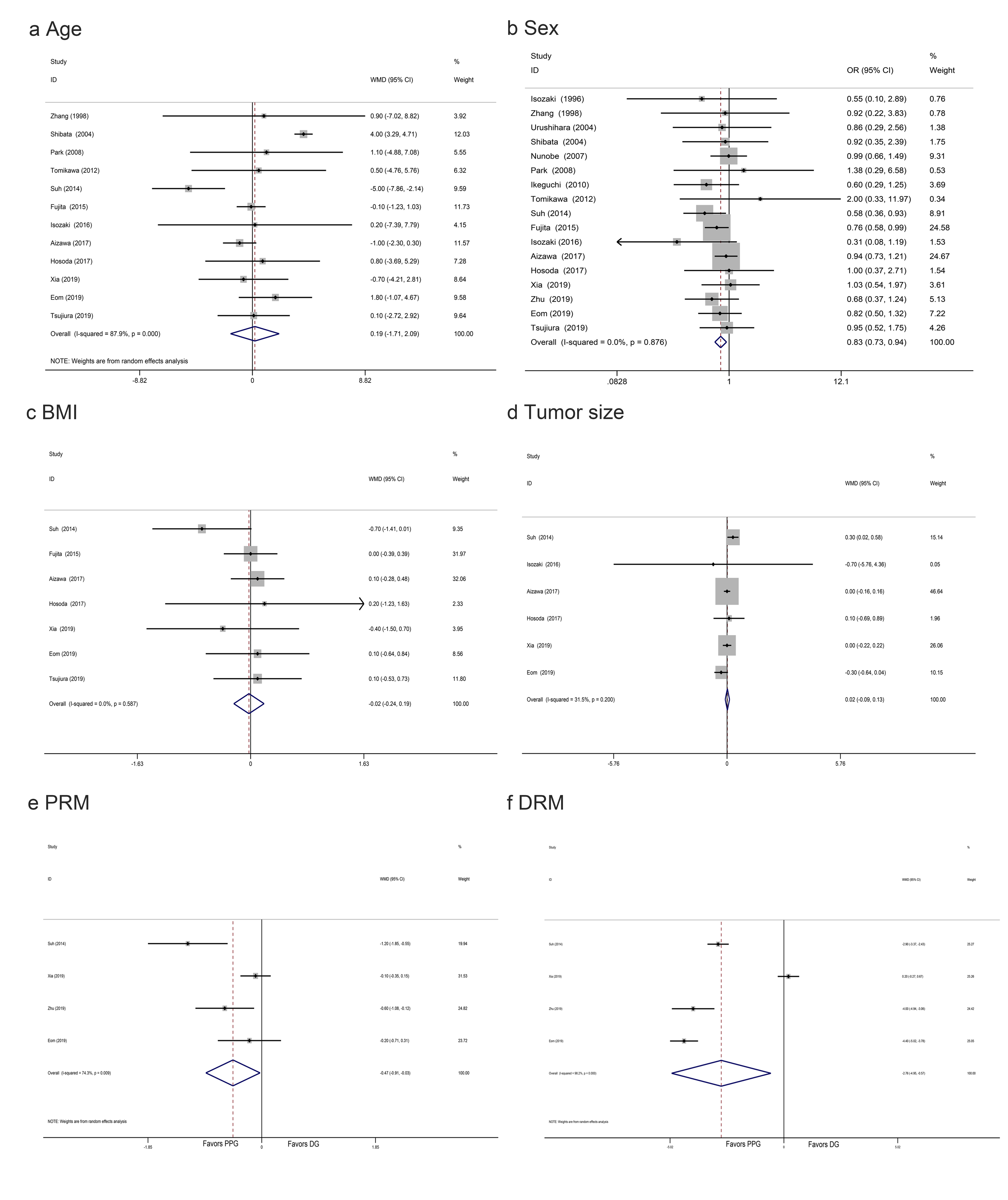

Supplement: Supplementary file 2 — Additional file 2. Forest plot of each outcome. (a) Age; (b) Sex; (c) BMI; (d) Tumor size; (e) PRM; (f) DRM; PRM, proximal resection margin; DRM, distal resection margin. [file 12957_2020_1910_MOESM2_ESM.tif]

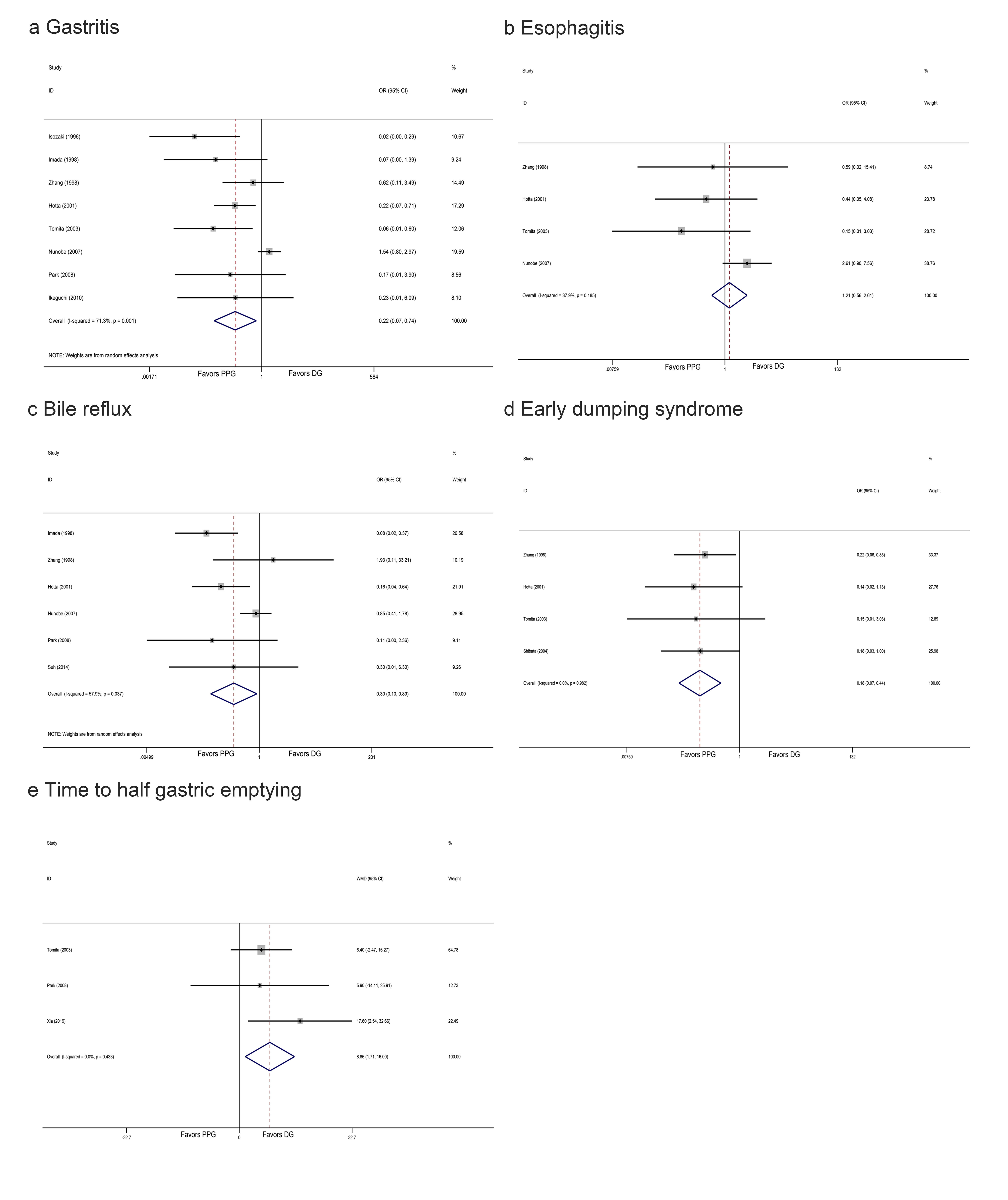

Supplement: Supplementary file 4 — Additional file 4. Forest plot of each outcome. (a) Gastritis; (b) Esophagitis; (c) Bile reflux; (d) Early dumping syndrome; (e) Time to half gastric emptying. [file 12957_2020_1910_MOESM4_ESM.tif]

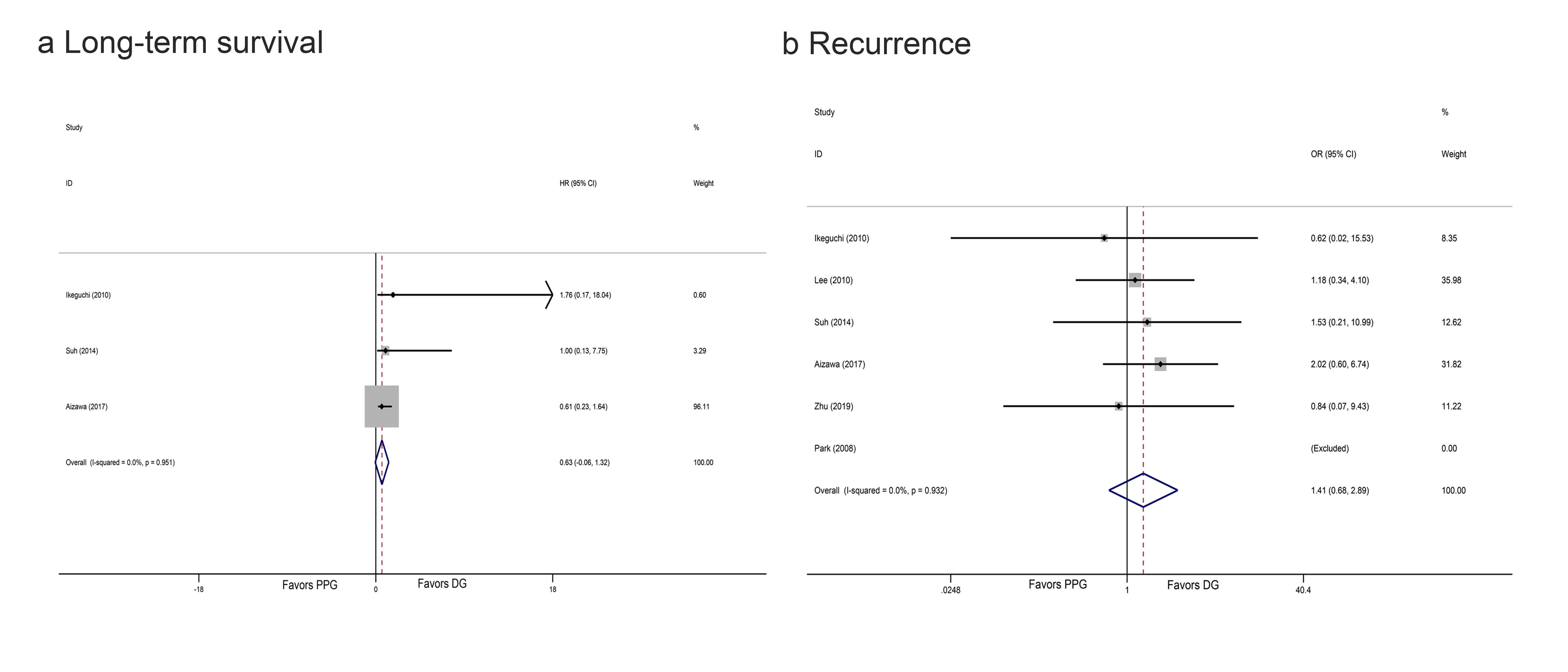

Supplement: Supplementary file 5 — Additional file 5. Forest plot of each outcome. (a) Survival rate; (b) Recurrence. [file 12957_2020_1910_MOESM5_ESM.tif]

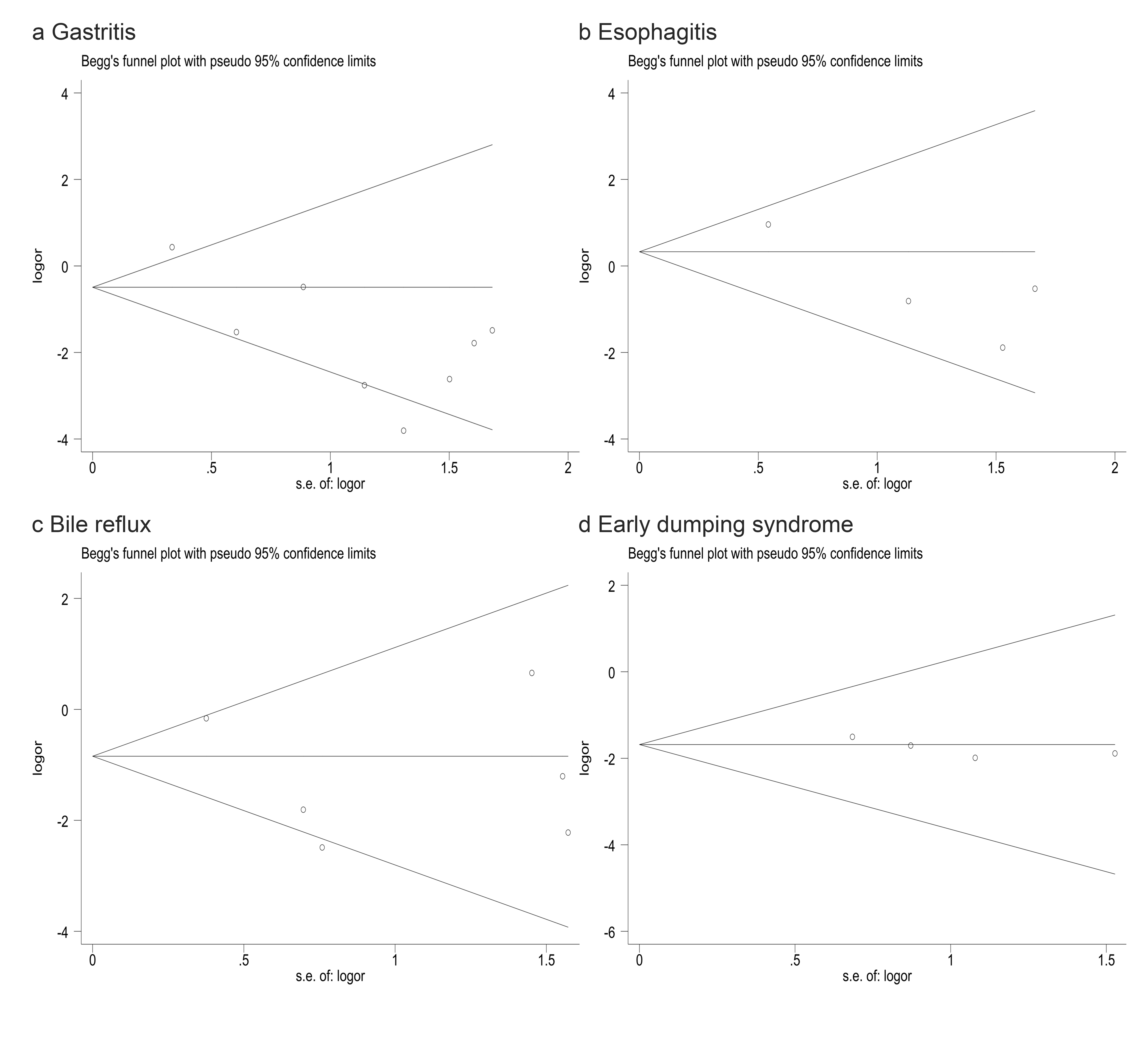

Supplement: Supplementary file 6 — Additional file 6. Funnel plots comparing (a) Gastritis, (b) Esophagitis, (c) Bile reflux, (d) Early dumping. [file 12957_2020_1910_MOESM6_ESM.tif]

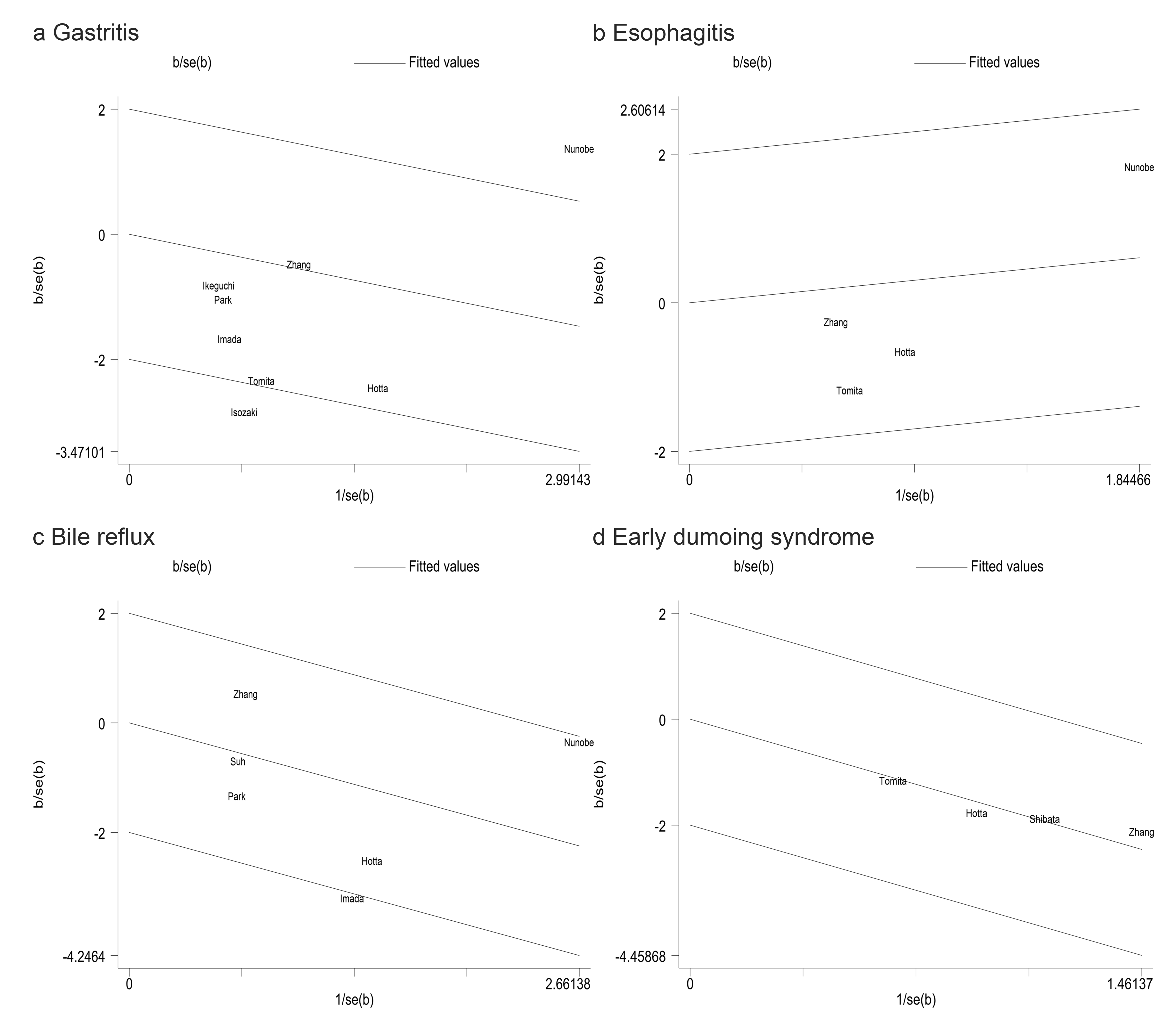

Supplement: Supplementary file 7 — Additional file 7. Galbraith plots comparing (a) Operative time, (b) Blood loss, (c) Number of retrieved lymph nodes, (d) Hospital day, (e) Postoperative complication, (f) Anastomotic leakage. [file 12957_2020_1910_MOESM7_ESM.tif]

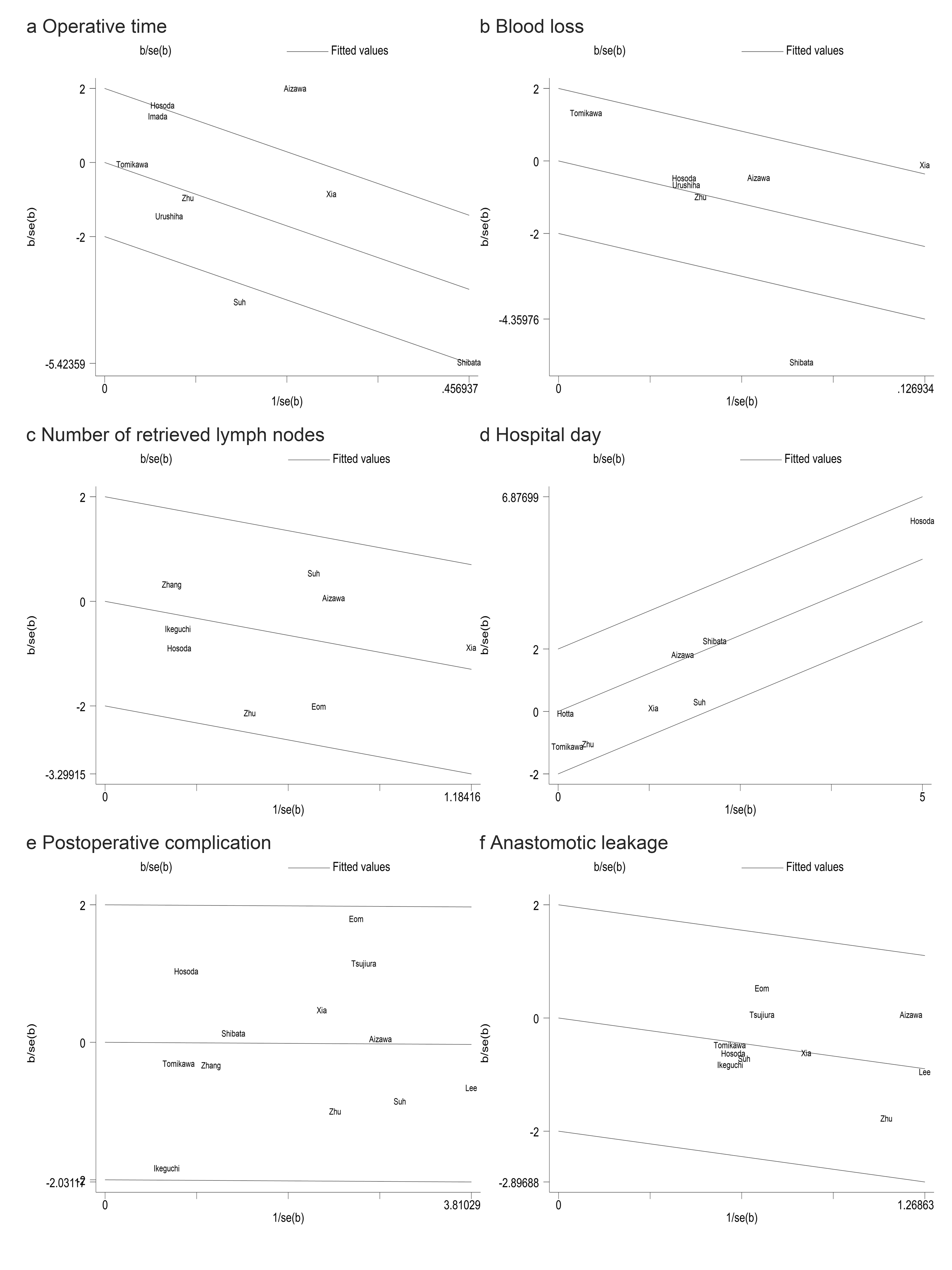

Supplement: Supplementary file 8 — Additional file 8. Galbraith plots comparing (a) Delayed gastric emptying, (b) Gallstone, (c) Total protein, (d) Albumin, (e) Hemoglobin, (f) Body weight loss. [file 12957_2020_1910_MOESM8_ESM.tif]

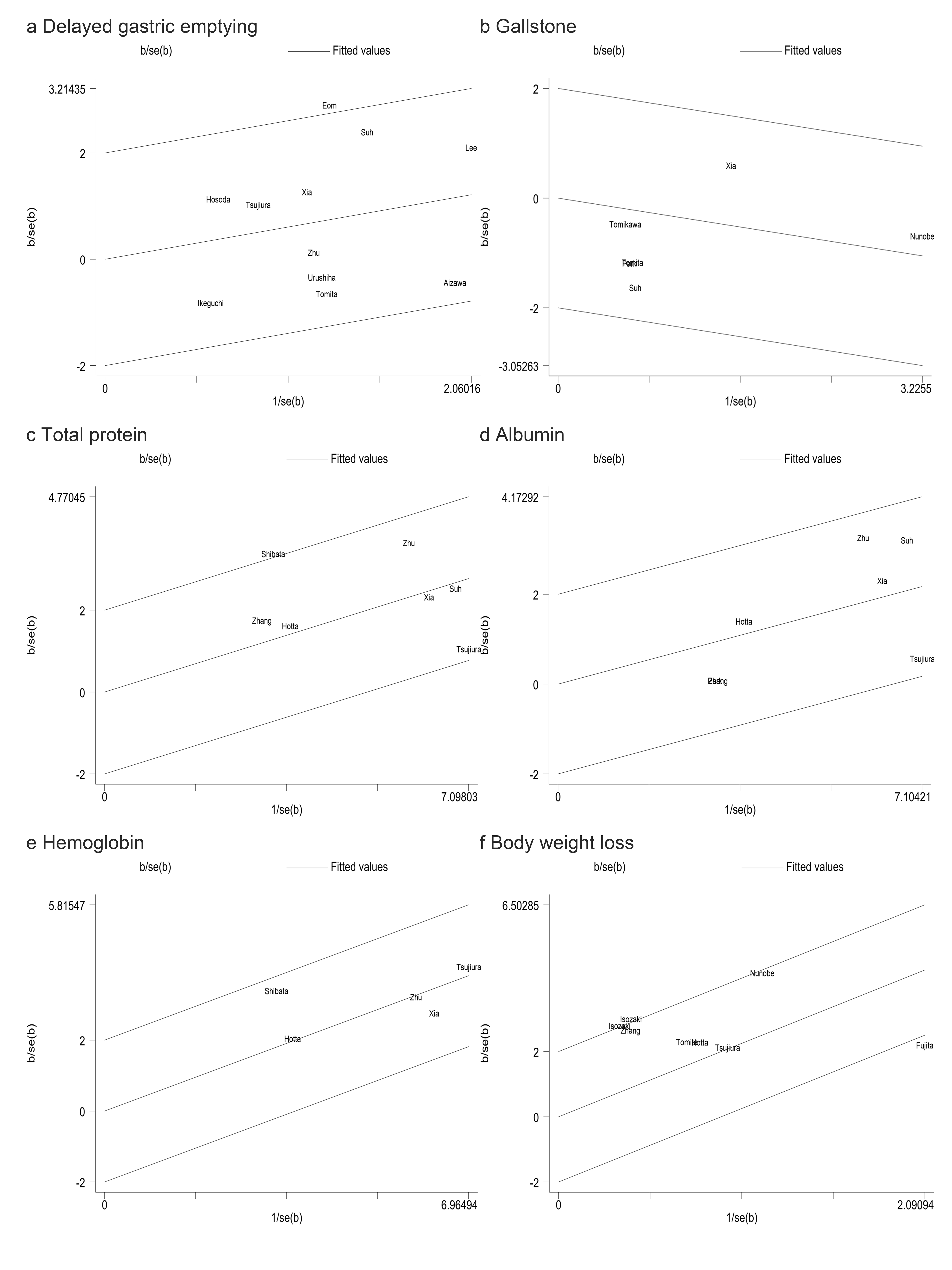

Supplement: Supplementary file 9 — Additional file 9. Galbraith plots comparing (a) Gastritis, (b) Esophagitis, (c) Bile reflux, (d) Early dumping syndrome. [file 12957_2020_1910_MOESM9_ESM.tif]
