# Supplementary material for: A comparison between pylorus-preserving and distal gastrectomy in surgical safety and functional benefit with gastric cancer: a systematic review and meta-analysis
Source: World J Surg Oncol. 2020 Jul 8;18:160. doi: 10.1186/s12957-020-01910-y (PMC7346397; doi:10.1186/s12957-020-01910-y)
Supplement: Supplementary file 3 — Additional file 3. Assessment of quality of studies. [file 12957_2020_1910_MOESM3_ESM.docx]

Table S2 Assessment of quality of studies.

| References | Selection | | | | Comparability | Outcome | | | Score |
| --- | --- | --- | --- | --- | --- | --- | --- | --- | --- |
|  | 1 | 2 | 3 | 4 | 5 | 6 | 7 | 8 |  |
| Suh et al. | * | * | * | * | ** | * | * | NA | 8 |
| Imada et al. | * | * | * | * | NA | * | NA | NA | 5 |
| Zhang et al. | * | * | * | * | * | * | NA | * | 7 |
| Hotta et al. | * | * | * | * | NA | * | NA | NA | 5 |
| Ikegucki et al. | * | * | * | * | * | * | * | * | 8 |
| Isozaki et al. | * | * | * | * | NA | * | NA | NA | 5 |
| Fujita et al. | * | * | * | * | ** | NA | NA | NA | 6 |
| Lee et al. | * | * | * | * | NA | * | * | NA | 6 |
| Nunobe et al. | * | * | * | * | NA | * | * | NA | 6 |
| Park et al. | * | * | * | * | NA | * | * | NA | 6 |
| Tomita et al. | * | * | * | * | NA | * | * | NA | 6 |
| Urushihara et al. | * | * | * | * | NA | * | * | NA | 6 |
| Aizawa et al. | * | * | * | * | ** | * | * | NA | 8 |
| Xia et al. | * | * | * | * | ** | * | NA | NA | 7 |
| Zhu et al. | * | * | * | * | ** | * | * | NA | 8 |
| Eom et al. | * | * | * | * | ** | * | * | NA | 8 |
| Tomikawa et al. | * | * | * | * | ** | NA | * | NA | 7 |
| Hosoda et al. | * | * | * | * | ** | NA | * | NA | 7 |
| Shibata et al. | * | * | * | * | ** | * | * | NA | 7 |
| Ikeguchi et al. | * | * | * | * | NA | * | * | * | 7 |
| Isozaki et al. | * | * | * | * | * |  | * | * | 7 |
| Tsujiura et al. | * | * | * | * | ** | * | * | NA | 8 |
